# Prophylactic Left Atrial Appendage Occlusion During Mitral Valve Repair in Patients Without Atral Fibrillation: A Meta‐Analysis of Time to Event Data

**DOI:** 10.1002/joa3.70355

**Published:** 2026-05-03

**Authors:** Ahmed Emara, Ameer Awashra, Mohamed S. Elgendy, Mohamed R. Murad, Mohamed Emara, Abubakar Nazir, Abdalhakim Shubietah, Michael Megaly, Vinayak N. Bapat

**Affiliations:** ^1^ Faculty of Medicine Al‐Azhar University Cairo Egypt; ^2^ Department of Medicine An Najah National University Nablus Palestine; ^3^ Faculty of Medicine Tanta University Tanta Egypt; ^4^ Department of Medicine Mercy Health‐The Jewish Hospital Cincinnati Ohio USA; ^5^ Department of Medicine Advocate Illinois Masonic Medical Center Chicago Illinois USA; ^6^ Integris Health Heart Hospital Oklahoma City Oklahoma USA; ^7^ Department of Cardiac Surgery, Minneapolis Heart Institute Foundation Abbott Northwestern Hospital Minneapolis Minnesota USA

**Keywords:** atrial fibrillation, left atrial appendage occlusion, mitral valve repair

## Abstract

**Background:**

Mitral valve repair (MVr) is effective for mitral regurgitation, but the benefit of prophylactic left atrial appendage occlusion (LAAO) in patients without prior atrial fibrillation (AF) remains unclear. This meta‐analysis aimed to assess the long‐term safety and efficacy of LAAO in this understudied population.

**Methods:**

We performed a meta‐analysis from four major databases until December 2025. Kaplan–Meier curves data were reconstructed and analyzed using Cox regression models and hazard ratios (HR) for thromboembolic events (mainly stroke). A random‐effects meta‐analysis was performed with R software to calculate risk ratios (RR), hazard ratios (HR), and mean differences (MD), all with 95% confidence intervals (CIs).

**Results:**

Three studies with 5048 patients were included. LAAO was associated with a significant reduction in thromboembolic risk at 5 years (HR 0.60, 95% CI 0.46 to 0.77). LAAO reduced in‐hospital stroke (RR 0.43, 95% CI 0.25 to 0.72) but increased postoperative AF (RR 1.17, 95% CI 1.09 to 1.26). No significant differences were observed in 30‐day mortality (RR 0.56, 95% CI 0.07 to 4.33) or hospital stay (MD –0.16 days, 95% CI – 0.48 to 0.16).

**Conclusions:**

Prophylactic LAAO during MVr in patients without AF may reduce thromboembolic events risk but appears to increase postoperative AF. Further randomized studies are warranted.

## Graphical abstract

In patients without atrial fibrillation undergoing mitral valve repair, prophylactic left atrial appendage occlusion significantly reduces long‐term thromboembolic events and early in‐hospital stroke, without increasing mortality, although it is associated with a higher incidence of postoperative atrial fibrillation.

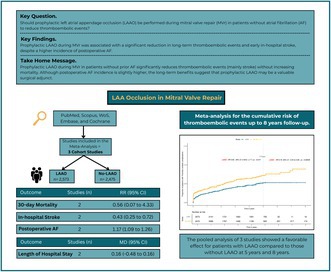

**Abbreviations**
AF: atrial fibrillation
LAA: left atrial appendage
LAAO: left atrial appendage occlusion
LOH: length of hospital stays
MVr: mitral valve repair

## 1 Introduction

Mitral valve repair (MVr) is usually associated with good outcomes for mitral regurgitation patients, but the occurrence of postoperative atrial fibrillation (AF) is reported to be greater than 30% and with a published annualized incidence of stroke of up to 1% [1, 2]. This highlights the need for strategies to mitigate this risk, particularly in patients without a prior history of AF. One proposed approach is prophylactic left atrial appendage occlusion (LAAO) during surgery [3].

The rationale for prophylactic LAAO is based on the fact that the left atrial appendage (LAA) is a common site of thrombus formation in patients with AF; thus, its closure may theoretically lower the risk of stroke [4]. Although certain studies indicate enhanced outcomes, others claim no significant difference compared to those who did not have LAAO [5, 6, 7]. These disparate findings underscore the need for additional research.

This meta‐analysis examined the effect of prophylactic LAAO performed at the time of MVr in patients without a history of AF. A pooled analysis of results from long‐term observational studies to provide insight into the impact of this intervention on the actual incidence of stroke and thromboembolism is useful for informing future surgical decision‐making.

## 2 Methods

PubMed, Scopus, Web of Science, and Cochrane databases were searched until December 2025 for studies evaluating the outcomes of patients undergoing MVr without recent or preoperative AF. The intervention was prophylactic LAAO, compared with MVr alone, and we reported our primary outcomes as thromboembolic events (mainly stroke). Full inclusions and exclusions criteria are presented in Table S1. We used the following search keywords: “Mitral valve repair,”, “Left atrial appendage occlusion,” and “No atrial fibrillation.” The full search strategy is presented in Table S2. A random‐effects model was conducted using DerSimonian‐Laird weighting, with risk ratio (RR) and mean difference (MD), both with 95% confidence intervals (CIs). Individual patient data were reconstructed from published Kaplan–Meier curves using the “IPDfromKM” R package and pooled for analysis. Treatment effects were assessed using Cox proportional hazards models, restricted mean survival time (RMST), restricted mean time lost (RMTL), landmark analysis, and time‐varying hazard estimation. RMST and RMTL estimates were obtained using regression‐based models with covariate adjustment where available, providing adjusted absolute measures of survival differences. Landmark and time‐varying analyses were used to evaluate temporal patterns of treatment effect. All data were analyzed in R software (V.4.4.2). Studies without extractable data for pairwise comparisons were not included in forest‐plot analyses but were included in Kaplan–Meier–based time‐to‐event analyses when applicable.

## 3 Results

The literature search identified 551 records. After removing 200 duplicates, 351 records were screened, of which 336 were excluded based on titles and abstracts. Full‐text review identified three studies meeting the inclusion criteria [5, 6, 7]. No additional studies were found through reference screening. The study selection process is summarized in the PRISMA flow diagram (Figure S1). Three retrospective cohort studies [5, 6, 7] were included in the final analysis comprising 5048 patients, 2573 (51%) in the LAAO group and 2475 (49%) in the no‐LAAO group (mean age; 68.04 ± 10.3 years, mean follow‐up: 6 years, mean CHA2DS2‐VASc: 2.75 ± 1.49). In the Tam et al., both device‐based and non‐device methods were used, although specific techniques were not consistently detailed [5]. In contrast, both Chikwe et al. and Ascaso et al. utilized internal surgical closure with sutures [6, 7]. Moreover, the three studies were “good quality” using Newcastle Ottawa Scale (NOS) which is presented in Table S3. Detailed summary and baseline characteristics of the included studies are outlined in Table 1.

**TABLE 1:** Summary and baseline characteristics of the included studies.

Characteristic	Tam et al., 2025		Chikwe et al., 2023		Ascaso et al., 2022	
	LAAO, *n* = 1875	No‐LAAO, *n* = 1875	LAAO, *n* = 431	No‐LAAO, *n* = 333	LAAO, *n* = 267	No‐LAAO, *n* = 267
Country	USA		USA		Canada	
LAAO technique	Device, non‐device and not specified		Internal surgical closure with sutures		Internal surgical closure with sutures	
Follow‐up, years	5 years		8 years		5 Years	
Age, *M* (SD)	71.12 (7.97)	70.82 (7.99)	62 (11.16)	57.33 (11.17)	58.63 (11.25)	59.23 (11.85)
Males, no. (%)	1026 (54.72)	1055 (56.26)	294 (68.2)	225 (67.5)	NA	NA
DM, no. (%)	342 (18.2)	328 (17.5)	21 (4.9)	18 (5.4)	9 (3.4)	8 (3.0)
Dyslipidemia, no. (%)	1285 (68.5)	1296 (69.1)	NA	NA	89 (33.3)	83 (31.1)
Dialysis, no. (%)	36 (1.9)	37 (2)	1 (0.23)	3 (0.9)	NA	NA
PVD, no. (%)	338 (18)	318 (17.0)	5 (1.2)	3 (1.2)	NA	NA
Hypertension, no. (%)	1478 (78.8)	1488 (79.4)	NA	NA	92 (34.5)	87 (32.6)
Previous CVA, no. (%)	371 (19.8)	358 (19.1)	3 (0.7)	3 (0.9)	13 (4.9)	10 (3.7)
CAD, no. (%)	1073 (57.2)	1076 (57.4)	NA	NA	29 (10.9)	28 (10.5)
Previous MI, no. (%)	138 (7.4)	154 (8.2)	6 (1.4)	8 (2.4)	NA	NA
CHA2DS2‐VASc, *M* (SD)	3 (1.48)	3 (1.48)	1.67 (0.74)	1.33 (0.74)	NA	NA
CPB time in min, *M* (SD)	NA	NA	113.3 (18.6)	132.67 (30.53)	71.6 (23.85)	73 (21.62)
Aortic clamping in min, *M* (SD)	NA	NA	74.67 (10.41)	89 (26.8)	54.6 (21.61)	(20.87) 54.6

Abbreviations: NA, not available: CAD, coronary artery disease; CPB, cardiopulmonary bypass; CVA, cerebrovascular accident; DM, diabetes mellitus; LAAO, left atrial appendage occlusion; *M*, mean; MI, myocardial infarction; No, number; PVD, peripheral vascular disease; SD, standard deviation.

**FIGURE 1:**
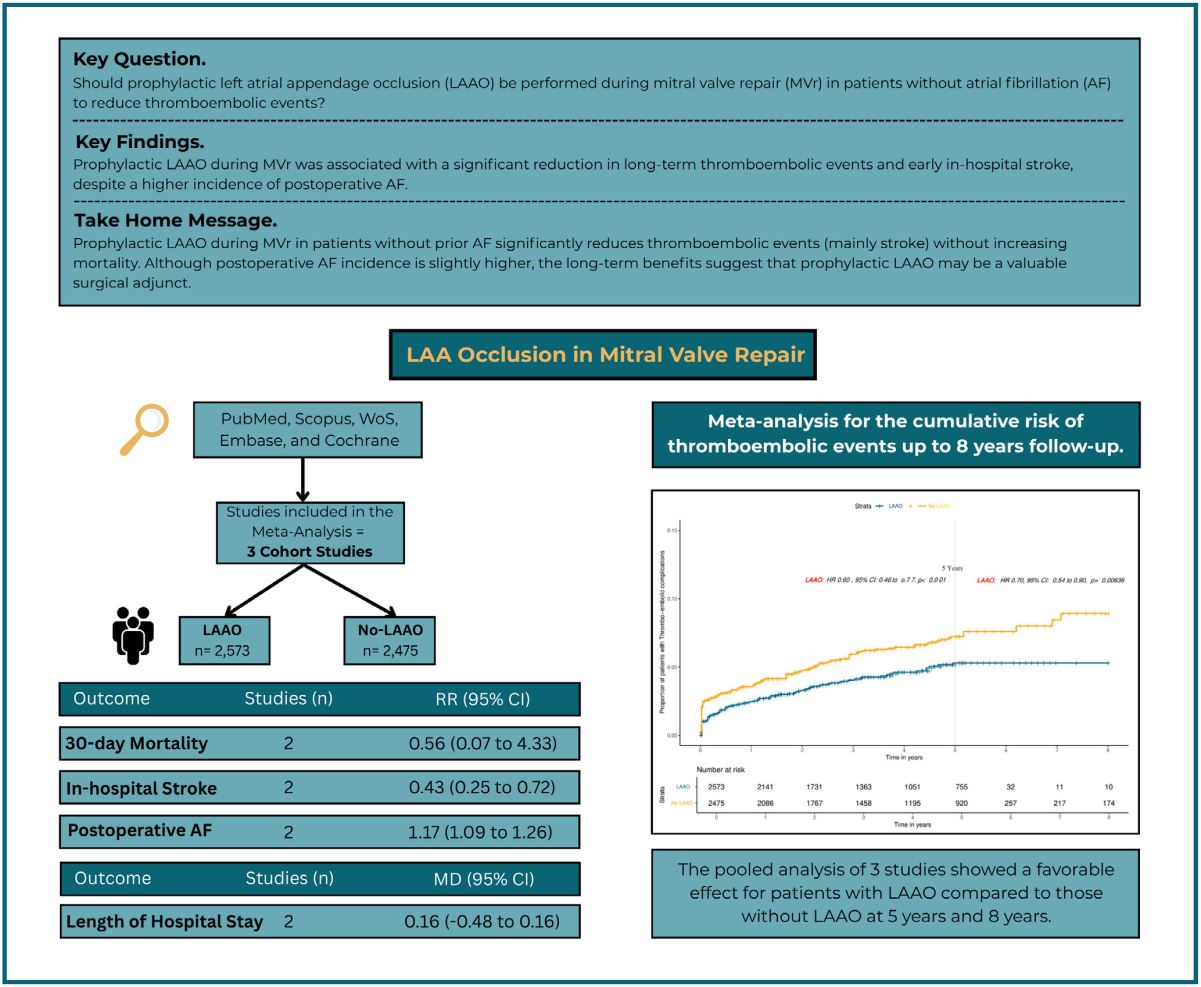
Kaplan–Meier curves reconstructed from published data comparing prophylactic left atrial appendage occlusion (LAAO) versus no LAAO during mitral valve repair in patients without atrial fibrillation. The curves illustrate cumulative thromboembolic risk over long‐term follow‐up (up to 8 years), with early separation observed during the perioperative and early post‐discharge period, followed by sustained divergence over time. Hazard ratios represent unadjusted estimates derived from reconstructed individual patient data.

**FIGURE 2:**
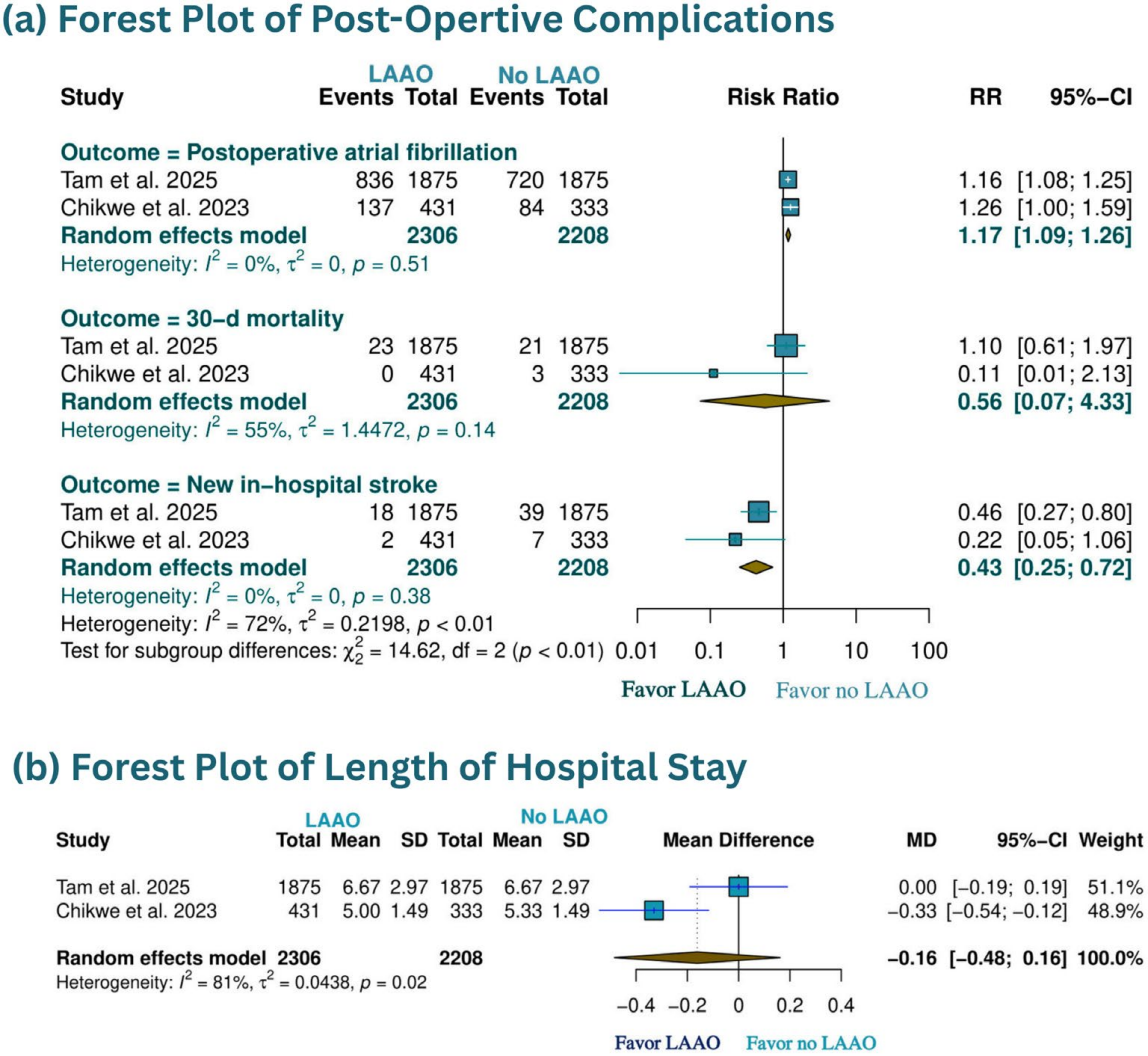
Forest plots comparing early postoperative outcomes between prophylactic left atrial appendage occlusion (LAAO) and no LAAO during mitral valve repair in patients without atrial fibrillation. Outcomes reflect events occurring during the index hospitalization or within 30 days postoperatively, including postoperative atrial fibrillation, 30‐day mortality, in‐hospital stroke, and length of hospital stay. Effect estimates are reported as risk ratios (RR) or mean differences (MD) with 95% confidence intervals (CIs).

We included 3 studies [5, 6, 7] for KM reconstruction of thromboembolic complications. The pooled IPD analysis showed a favorable effect for patients with LAAO compared to whom without LAAO at 5 and 8 years (HR 0.60, 95% CI 0.46 to 0.77, *p* < 0.001; HR 0.70, 95% CI 0.54 to 0.90, *p* = 0.0063) (Figure 1). To further explore the temporal distribution of treatment effect and address potential early‐event bias, we performed RMST and landmark analyses. In this pooled analysis of three studies (*N* = 4844), LAAO was associated with a significant improvement in 5‐year RMST compared with no‐LAAO (4.827 vs. 4.752 years; difference 0.075 years [0.9 months]; 95% CI 0.022 to 0.128, *p* = 0.0054) (Figure S2). Consistent findings were observed for RMTL, with LAAO showing 0.075 years less time lost (95% CI 0.540 to 0.900, *p* = 0.0056) (Figure S3). A 30‐day landmark analysis revealed no significant difference in post‐landmark RMST (HR 0.87, 95% CI 0.63 to 1.18, *p* = 0.362), suggesting that the observed benefit was largely driven by early postoperative events (Figure S4). However, time‐varying hazard ratio analysis demonstrated a progressively strengthening protective effect over time (HR 0.85 at 1 year, 0.78 at 2 years, 0.74 at 3 years, 0.71 at 4 years, and 0.69 at 5 years) (Figure S5). Also, the random‐effect meta‐analysis showed no difference between two groups in 30‐day mortality (RR 0.56, 95% CI 0.07 to 4.33), or length of hospital stay (LOH) (MD – 0.16 days, 95% CI – 0.48 to 0.16) (Figure 2). However, LAAO significantly decreased new in‐hospital stroke (RR 0.43, 95% CI 0.25 to 0.72), despite increasing the incidence of postoperative AF (RR 1.17, 95% CI 1.09 to 1.26) (Figure 2).

## 4 Discussion

The concomitant surgical LAAO has been shown to be safe in cardiac surgery patients with AF [3]. Similarly, the beneficial effect of LAAO in all cardiac surgery patients without a prior AF has recently been proven by a meta‐analysis by Zhao et al. [8]. In their analysis, LAAO was associated with a 58% decrease of early thromboembolic events with a 52% decrease at 6 years. In our analysis on MVr patients without a prior AF, LAAO was associated with a sustained 30% reduction at 8 years and a 40% relative reduction at 5 years.

Additionally, our analysis demonstrated that LAAO decreased rates of new in‐hospital stroke compared to no‐LAAO (0.87% vs. 2.1%), providing consistency with a short‐term clinical benefit specifically for patients with a stroke risk. With these positive results, LAAO might be appropriate in patients with no known AF and has already been applied in clinical practice [9]. Additional time‐to‐event analyses using RMST and landmark methods suggest that the observed benefit of LAAO is largely driven by early postoperative risk reduction. The 30‐day landmark analysis showed no significant difference beyond this period, supporting the predominance of early effects. However, time‐varying hazard analysis demonstrated a gradual divergence over time, indicating that a modest long‐term benefit cannot be excluded. These findings should be interpreted cautiously given the observational design and lack of fully adjusted individual‐level data.

While LAAO seems effective in preventing early thromboembolic complications, we found an increased risk in the LAAO group (42.2% vs. 36.4%) for postoperative AF. This aligns with findings from the meta‐analysis by Zhao et al., showing similar results across all cardiac surgery patients [8], with a randomized controlled trial (RCT)—reported in that analysis—having also a higher AF incidence with LAAO (47.3% vs. 38.2%) [10]. As LAAO involves cardiac mobilization, which can lead to ischemia in the left atrial tissue, the technique used may not always fully isolate the LAA electrically. Since the LAA is the most flexible part of the left atrium, it helps prevent pressure spikes, which could increase the risk of postoperative AF [3, 11]. Several mechanistic explanations may account for this observation. First, intraoperative atrial manipulation inherent to LAAO, such as traction, suturing, or stapling, can induce local inflammation and ischemia, predisposing to transient electrophysiological instability within atrial tissue [3, 8]. Second, atrial compliance may be altered after appendage exclusion. The LAA contributes to left atrial reservoir and conduit functions; its removal can increase left atrial pressure and strain, which in turn promotes atrial stretch–induced depolarization and triggers AF [11]. Third, in cases where electrical isolation of the LAA is incomplete, residual electrically active tissue may serve as a potential arrhythmogenic focus, facilitating reentrant circuits and postoperative AF. Finally, acute changes in atrial geometry and stiffness following surgical closure, particularly with suture techniques, can disturb atrial mechano‐electric coupling and conduction heterogeneity, further increasing AF susceptibility. Device‐based or surgical LAAO carries long‐term risks, including incomplete exclusion or recanalization, which can leave a thrombogenic LAA stump [6, 7]. Techniques such as purse‐string ligation or external clips may result in residual communication in 15%–60% of patients, creating areas of stagnant flow that promote thrombus formation [6]. Anatomical variability may also prevent full occlusion with clips or staples, leaving trabeculated tissue exposed. These factors highlight the importance of achieving complete mechanical exclusion to optimize the safety and efficacy of LAAO [6].

Furthermore, 30‐day mortality and LOH did not significantly differ between the studies included. Specifically, the Ascaso study showed similar 5‐year mortality rates between the two groups [7], while the Tam study found no significant difference in survival rates at both 30 days and 5 years [5]. These studies indicate that LAAO does not appear to significantly affect short‐term or long‐term mortality outcomes, indicating an acceptable safety profile. This study has several limitations. First, although time‐to‐event analyses including RMST, RMTL, landmark analysis, and time‐varying hazard modeling were performed, these estimates were derived from reconstructed survival data at the study level rather than individual patient data. Therefore, adjustment for patient‐level covariates was not feasible, and potential residual confounding across studies cannot be fully excluded. Second, despite the use of complementary survival metrics, the findings should be interpreted as associative rather than definitively causal, particularly given the observational nature of the included studies and potential differences in baseline characteristics between groups. Third, data on postoperative antithrombotic therapy were not available across the included studies. Variations in antiplatelet or anticoagulation regimens after MVr could have influenced thromboembolic outcomes, and the absence of these data represents an important limitation of our analysis. Fourth, the included studies used different LAAO techniques; Tam et al. [5] used both device‐ and non‐device‐based methods, whereas Chikwe and Ascaso [6, 7] used suture‐based closure. This variation may influence thromboembolic and AF outcomes. Due to limited data, subgroup analyses by closure method were not feasible, representing an important limitation. Future randomized trials or studies reporting adjusted survival analyses are needed to confirm these results.

## 5 Conclusion

Our meta‐analysis suggests that prophylactic LAAO during MVr in patients without prior AF may lower long‐term thromboembolic risk and early in‐hospital stroke, but its link to increased incidence of postoperative AF highlights the need for large‐scale RCTs to refine surgical approaches.

## Author Contributions

A.E.: validation, conceptualization, project administration, investigation, writing – original draft. A.A.: investigation, writing – original draft. M.S.E.: material preparation, investigation, visualization, writing – original draft, manuscript drafting. M.R.M.: investigation, formal analysis, writing – original draft. M.E., A.Z., and A.S.: investigation, writing – original draft. M.M.: writing – review and editing, supervision. V.N.B.: writing – review and editing, supervision. No generative AI tools or paper mills were used in the preparation of this manuscript. All authors have read and approved the final version of the manuscript.

## Funding

The authors have nothing to report.

## Disclosure

We affirm that this work is original, has not been published previously, and is not under consideration for publication elsewhere. None of the paper's contents has been previously published. All authors have read and approved the manuscript. We also provide full disclosure of any relationships with industry.

## Ethics Statement

Ethical approval is not required for this review article.

## Consent

The authors have nothing to report.

## Conflicts of Interest

The authors declare no conflicts of interest.

## Supporting information


**Table S1:** Full inclusion and exclusion criteria.
**Table S2:** Search strategy and literature search results.
**Table S3:** Newcastle Ottawa Scale (NOS) for quality assessment of observational cohort studies.
**Figure S1:** PRISMA flow chart for the systematic search and selection process.
**Figure S2:** Kaplan Meier Curve of Adjusted RMST.
**Figure S3:** Kaplan Meier Curve of Adjusted RMTL.
**Figure S4:** Kaplan Meier Curve of Land mark analysis time 30 days.
**Figure S5:** Time varying Hazard Ratio.

## Data Availability

This study did not involve the creation or analysis of any new data. Therefore, data sharing is not applicable.
