# Supplementary material for: Prophylactic Left Atrial Appendage Occlusion During Mitral Valve Repair in Patients Without Atral Fibrillation: A Meta‐Analysis of Time to Event Data
Source: J Arrhythm. 2026 May 3;42(3):e70355. doi: 10.1002/joa3.70355 (PMC13136685; doi:10.1002/joa3.70355)
Supplement: Supplementary file 1 — Table S1: Full inclusion and exclusion criteria. Table S2: Search strategy and literature search results. Table S3: Newcastle Ottawa Scale (NOS) for quality assessment of observational cohort studies. Figure S1: PRISMA flow chart for the systematic search and selection process. Figure S2: Kaplan Meier Curve of Adjusted RMST. Figure S3: Kaplan Meier Curve of Adjusted RMTL. Figure S4: Kaplan Meier Curve of Land mark analysis time 30 days. Figure S5: Time varying Hazard Ratio. [file JOA3-42-e70355-s001.pdf]

Supplementary Materials

Prophylactic Left Atrial Appendage Occlusion During Mitral Valve Repair in Patients Without Atral Fibrillation: A Meta-Analysis of Time to Event Data

Authors.

Ahmed Emara, MD; Ameer Awashra; MD; Mohamed S. Elgendy, MD; Mohamed R. Murad, MD; Mohamed Emara, MD; Abubakar Nazir, MD; Abdalhakim Shubietah; MD; Michael Megaly, MD MS; Vinayak N. Bapat, MD MS

Supplementary table 1. Full inclusion and exclusion criteria.

**Inclusion criteria**	Studies were eligible if they included adult patients ≥ 18 years old undergoing surgical mitral valve repair who were in sinus rhythm and had no documented history of preoperative or prior atrial fibrillation. Eligible studies compared prophylactic left atrial appendage occlusion (LAAO) performed concomitantly with mitral valve repair versus mitral valve repair alone. Both device-based and surgical (suture-based) LAAO techniques were considered acceptable. Studies were required to report at least one relevant clinical outcome, including thromboembolic events (primarily stroke), postoperative atrial fibrillation, mortality, or length of hospital stay, with sufficient data to allow effect estimation or time-to-event analysis. All randomized controlled trials and observational cohort studies with a comparator group, adequate follow-up, and published in the English language were included. All included studies were published in a peer reviewed-journal.
**Exclusion criteria**	Studies were excluded if they enrolled patients with pre-existing or recent atrial fibrillation, included mitral valve replacement rather than repair, lacked a control group, or did not report relevant clinical outcomes. Case reports, case series without comparators, reviews, editorials, conference abstracts, non-English publications, and studies with overlapping populations or insufficient data for extraction were also excluded.

Supplementary Table 2 Search strategy and literature search results.

Database	Filters	Access Date	Search Strategy	No of Results
**PubMed**	All Fields	29/12/2025	("Mitral Valve Repair" OR MVR OR "Mitral Valve Plasty" OR "mitral valve reconstruction" OR "mitral repair") AND ("Left Atrial Appendage" OR "left atrial appendage" OR LAA) AND (occlusion OR exclusion OR amputation OR closure OR ligation)	**117**
**Cochrane**	All Text	29/12/2025		**7**
**Scopus**	Title, abstracts, and keywords	29/12/2025		**310**
**Web of Science**	All Fields	29/12/2025		**117**
**Total**				**551**

Supplementary Table 3. Newcastle Ottawa Scale (NOS) for quality assessment of observational cohort studies.

Study	Selection				Comparability	Outcome			Quality
	Representativeness of the exposed cohort	Selection of the non-exposed cohort	Ascertainment of exposure	outcome of interest was not present at start of study		Assessment of outcome	Was follow-up long enough for outcomes to occur	Adequacy of follow-up of cohorts	
**Tam et al., 2025**	*	*	*	*	**	*	*	*	Good quality
**Chikwe et al., 2023**	*	*	*	*	**	*	*	*	Good quality
**Ascaso et al., 2022**	*	*	*	*	**	*	*	*	Good quality

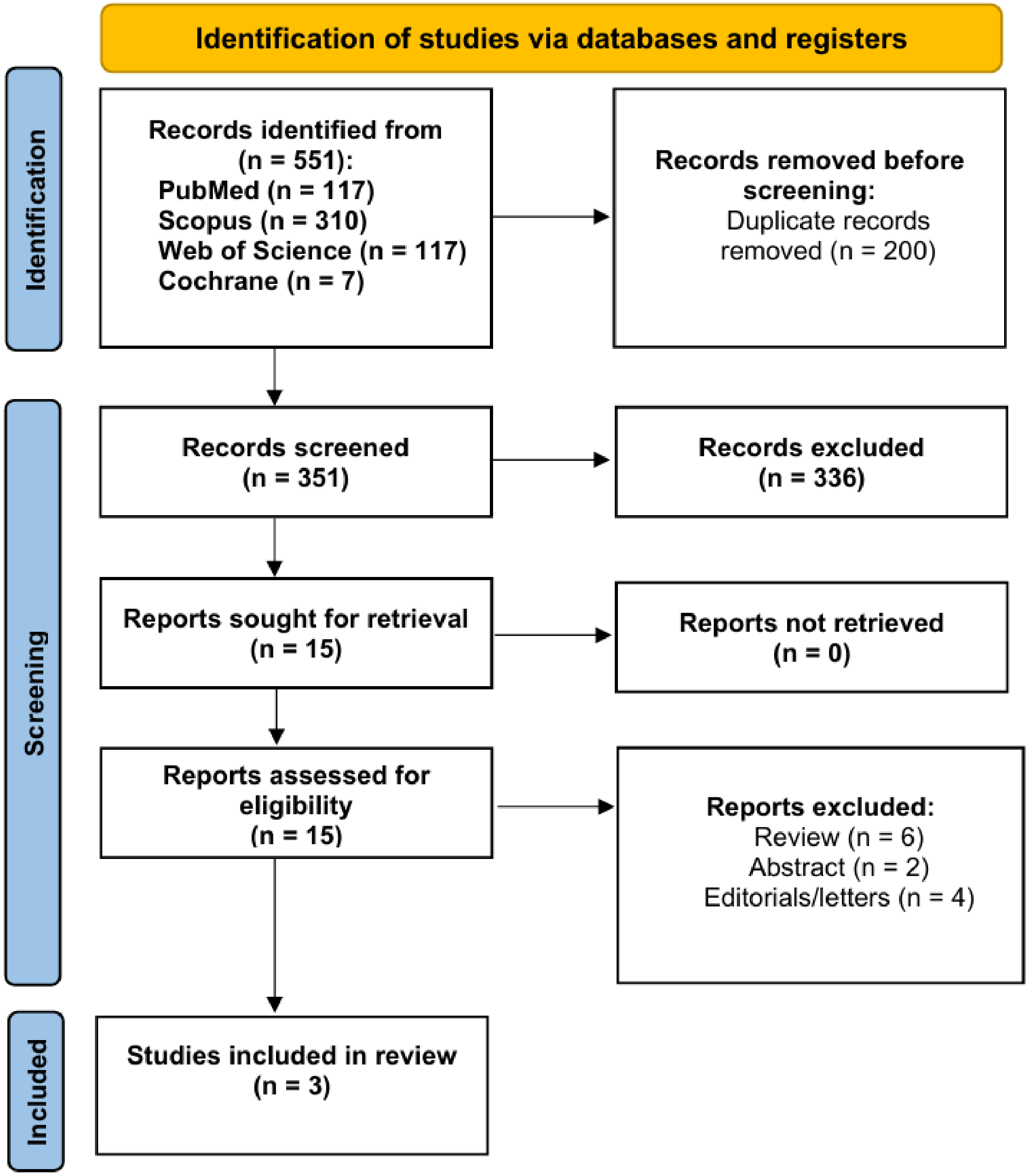

Supplementary Figure 1. PRISMA flow chart for the systematic search and selection process.

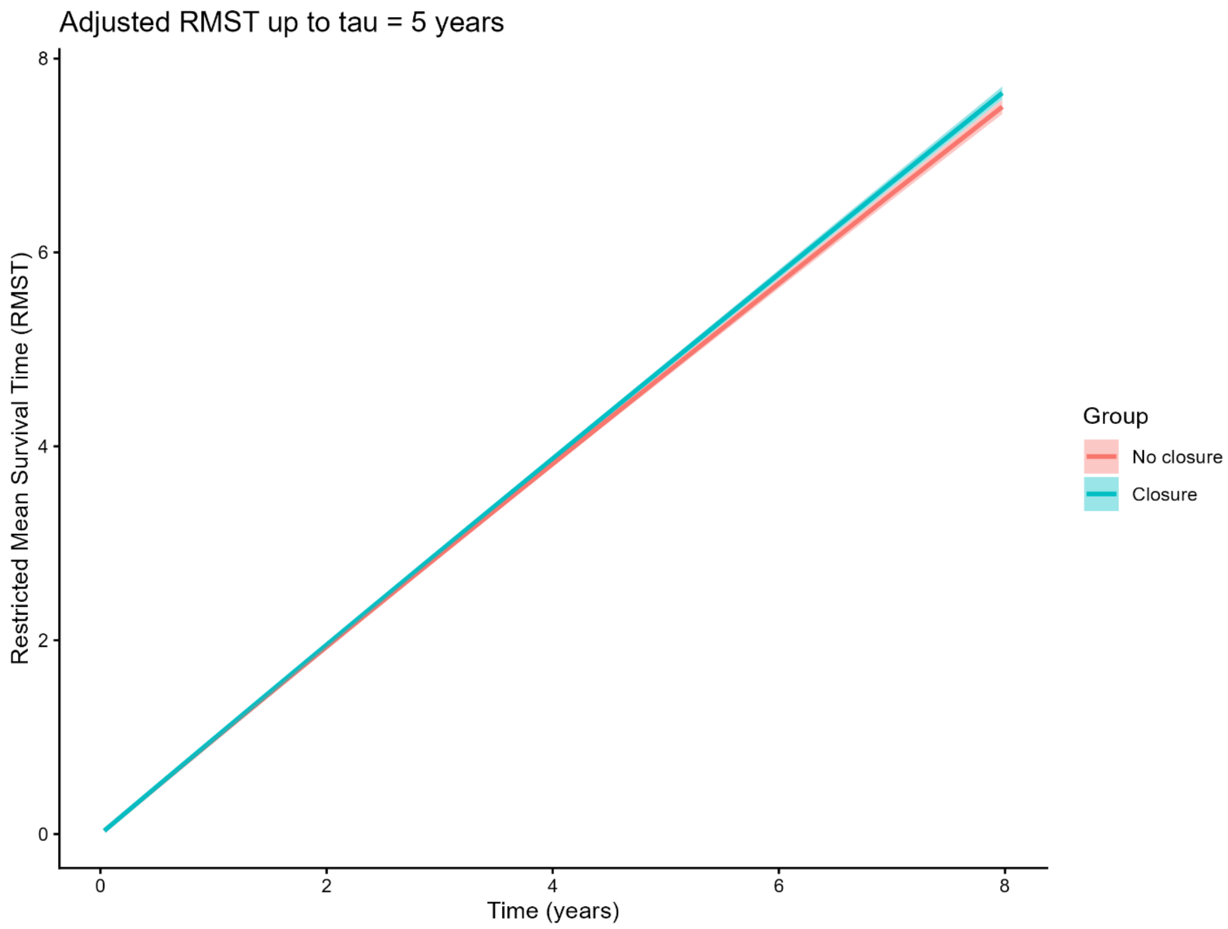

Supplementary Figure 2. Kaplan Meier Curve of Adjusted RMST.

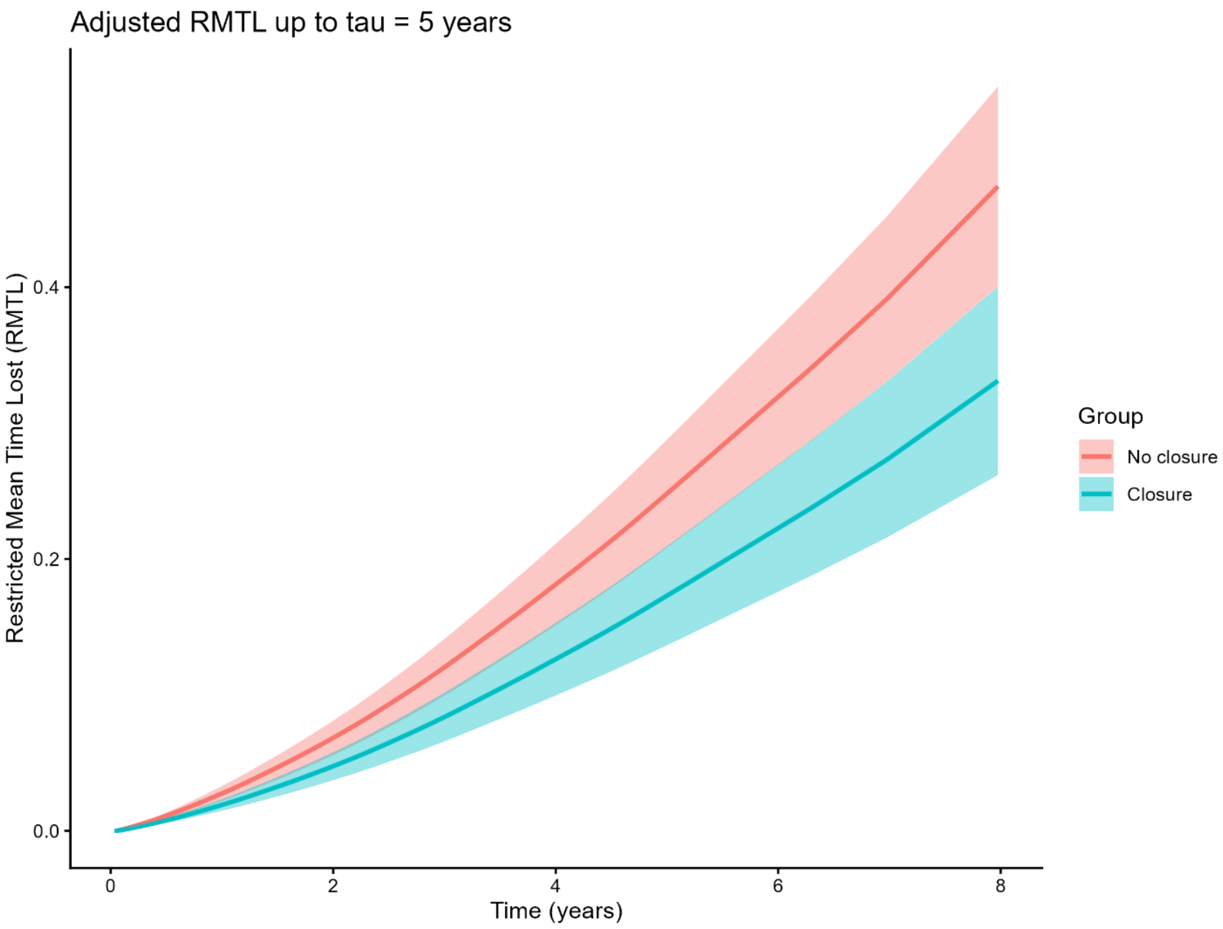

Supplementary Figure 3. Kaplan Meier Curve of Adjusted RMTL.

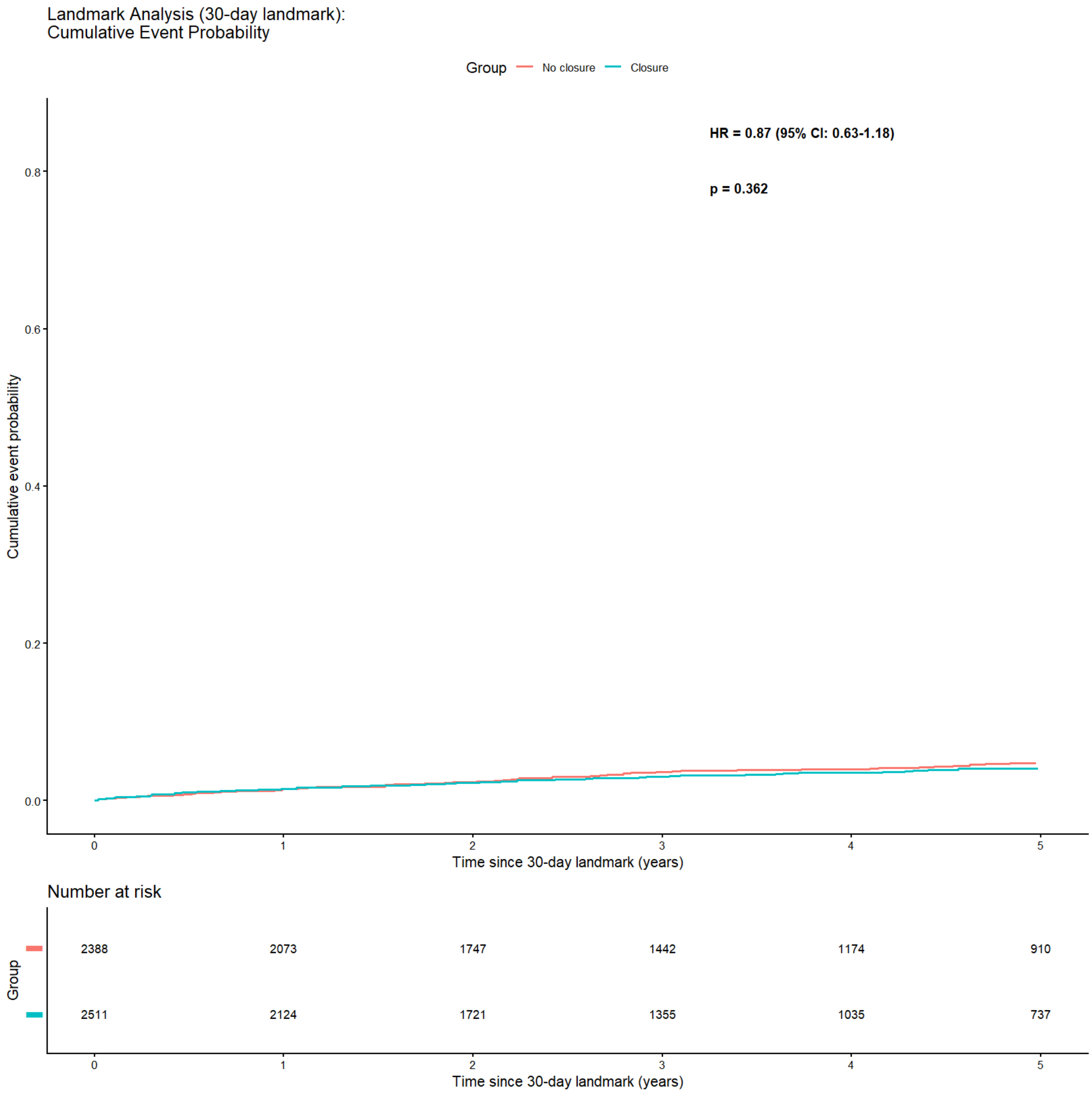

Supplementary Figure 4. Kaplan Meier Curve of Land mark analysis time 30 days.

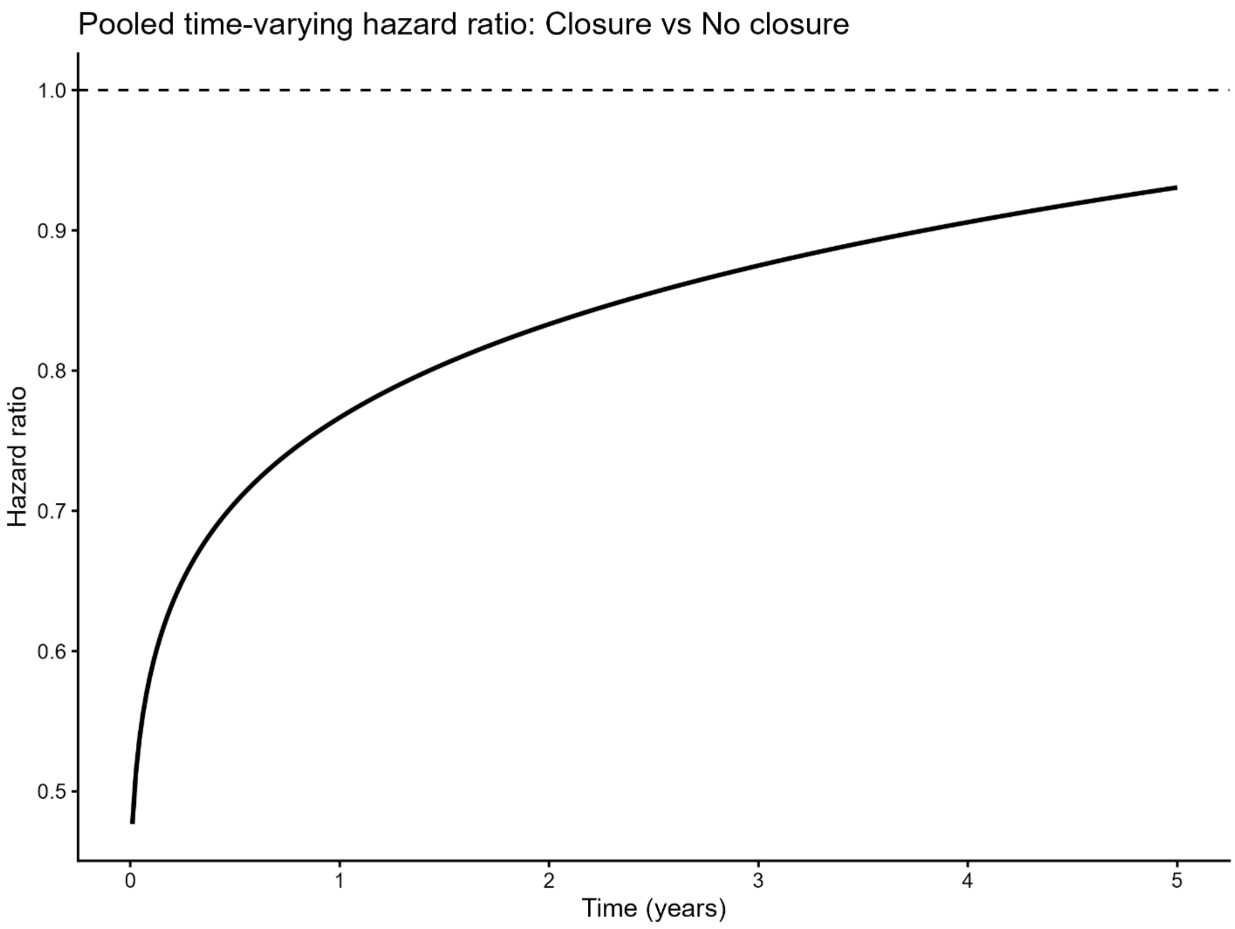

Supplementary Figure 5. Time varying Hazard Ratio.
